# Application of Bovine Pericardium and Expanded Polytetrafluoroethylene Patches in Tricuspid Valvuloplasty after Cardiac Surgery

**DOI:** 10.31083/j.rcm2505188

**Published:** 2024-05-23

**Authors:** Shuo Xiao, Qiuji Wang, Dou Fang, Zhenzhong Wang, Yingjie Ke, Zhaolong Zhang, Yuxin Li, Lishan Zhong, Huanlei Huang

**Affiliations:** ^1^Medical school, South China University of Technology, 510006 Guangzhou, Guangdong, China; ^2^Department of Cardiac Surgery, Guangdong Cardiovascular Institute, Guangdong Provincial People's Hospital (Guangdong Academy of Medical Sciences), Southern Medical University, 510080 Guangzhou, Guangdong, China

**Keywords:** tricuspid valvuloplasty, leaflet augmentation, bovine pericardium, expanded polytetrafluoroethylene, patch

## Abstract

**Background:**

Leaflet augmentation is often required to correct an inadequate leaflet size due to leaflet thickening, contracture and junctional fusion in patients with tricuspid valve regurgitation (TR) after left-side valve surgery (LSVS). However, the ideal material for leaflet augmentation remains controversial. This article aims to compare the medium- and long-term results of tricuspid valve repair with bovine pericardium (BP) and expanded Polytetrafluoroethylene (ePTFE) patches for the augmentation of tricuspid leaflets and to compare the durability of the two materials.

**Methods:**

From January 2015 to April 2023, a total of 69 patients with severe isolated TR underwent tricuspid valvuloplasty (TVP) by leaflets augmentation with patches in our institute. According to the different types of patches, they were divided into the BP group (n = 44) and the ePTFE group (n = 25).

**Results:**

There were 3 perioperative deaths (4.3%), one case was due to low cardiac output syndrome in the BP group, and 2 cases were due to acute respiratory dysfunction syndrome and low cardiac output syndrome in the ePTFE group, respectively. Before discharge, the area of the TR jet on echocardiography decreased from 23.5 ± 9.1 to 4.2 ± 3.4 ${\mathrm{cm}}^{2}$. One case in each group was found to have increased blood flow velocity at the tricuspid orifice. After discharge, one patient in each group underwent repeat TVP, in the BP group because of shortened chordae and in the ePTFE group because of calcification of the patch. During the entire follow-up period, there were 7 cases of severe TR (10.1%), 5 in the BP group and 2 in the ePTFE group, a total of 5 cases of tricuspid stenosis (7.2%), 4 in the BP group and 1 in the ePTFE group, and a total of 6 deaths (8.7%), 5 in the BP group and 1 in the ePTFE group. Transthoracic ultrasound in a patient with tricuspid stenosis suggests stiff leaflet movement and poor motion.

**Conclusions:**

Leaflet patch enlargement can be safely used in tricuspid valve repair, but BP patches carry a risk of reduced flexibility and stiffness of movement, and ePTFE patches carries a risk of calcification.

## 1. Introduction

It has been reported that the prevalence of significant-moderate or severe tricuspid valve regurgitation (TR) is as high as 0.55% [1], while the incidence of long-term significant TR after left-side valve surgery (LSVS) is approximately 27% [2]. TR is a public health crisis, untreated isolated TR can significantly affect survival [3, 4, 5].

According to the ACC/AHA guidelines for the management of heart valve diseases, tricuspid valve surgery can be considered in patients with severe TR unresponsive to medical therapy and considered in asymptomatic patients with severe TR with evidence of right ventricular dilatation or systolic dysfunction. And should be performed as early as possible to avoid irreversible right ventricular dysfunction and organ failure [6]. Compared with valve replacement, valve repair in the tricuspid valve position is significantly more advantageous because the tricuspid valve has a slow blood flow rate, mechanical valves are more likely to form thrombosis, and often require higher intensity of long-term anticoagulation therapy. Unfortunately, the durability of the bioprosthetic valve in the tricuspid position is poor.

Isolated TR after LSVS for rheumatic heart disease (RHD) is a special type and accounts for the majority [7, 8], and often presents with significant cardiac remodelling, significant enlargement of the right heart, and organic lesions of the valve leaflets. In addition, after a long course of disease, some patients diagnosed with functional TR preoperatively were often found to have organic lesions of the leaflets during surgery [9]. Satisfactory results of tricuspid valvuloplasty (TVP) cannot be achieved by implanting a ring alone under the circumstance, and further valve leaflet augmentation is often required to restore normal coaptation [10]. We have reported a novel minimally invasive tricuspid valve repair technique by leaflet augmentation with patch on beating heart [11], however, it is unclear whether there is difference in medium- to long-term effects between pericardial patch and expanded polytetrafluoroethylene (ePTFE) (0.4 mm, GORE-TEX) patch (Lot number: 27101280, W.L. GORE & ASSOCIATES, INC., Flagstaff, AZ, USA). The pericardial patch is reported to have a high tendency for calcification within the first 5–8 years [12, 13, 14], and ePTFE is reported to be prone to avulsion in aortic valve repair [15]. The short-term and ultimate long-term durability of the patch will affect the prognosis of patients. Therefore, we conducted this study to analyse the durability of both patches in TVP with leaflet augmentation.

## 2. Materials and Methods

### 2.1 Study Patients

The clinical data of 100 patients, with isolated TR following cardiac surgery, who underwent TVP in our institute from January 2015 to April 2023 were analysed. Thoracic ultrasound showed severe TR in all patients, and most patients had right heart enlargement. We excluded those patients who had not received leaflet enlargement, had no previous history of heart surgery and accompanied with left-side valve surgery at the same time. Finally, 69 out of 100 patients who underwent leaflet augmentation were included in the study. Of the 69 patients who underwent patch expansion of the leaflet, 25 patients were implanted with ePTFE patches and 44 with bovine pericardium (BP) patches. During the surgery, except for different patches, both groups underwent the same surgical procedure. Among the 69 patients with a history of heart surgery (about 17.7 ± 6.9 years ago), 27 patients underwent double valve replacement, 40 patients underwent mitral valve replacement, 1 patient underwent Bentall surgery, and 1 patient underwent ventricular septal defect repair, respectively. Sixteen patients treated with annuloplasty ring alone (performed as a concomitant procedure) in the previous surgery underwent Re-do tricuspid valve surgery because of insufficient effective tricuspid leaflet area. There were 60 patients suffering from atrial fibrillation, and 2 patients underwent pacemaker implantation procedure before the TVP surgery. The perioperative information was shown in Table 1.

**Table 1.** **Patient characteristics**.

Variable	Entire study population	Bovine pericardial group	ePTFE group	t/χ^2^	*p*
	N = 69	N = 44	N = 25		
Age (y)	55.9 ± 8.6	55.9 ± 7.8	55.8 ± 10.2	0.078	0.938
Male (male, n)	13 (18.8%)	9 (20.5%)	4 (16.0%)	0.018	0.893
BMI (kg/$m^{2}$)	22.0 ± 2.6	22.1 ± 2.7	21.8 ± 2.5	0.424	0.673
Diabetes	2 (2.9%)	1 (2.3%)	1 (4.0%)	0.169	0.681
Hypertension	18 (26.1%)	14 (31.8%)	4 (16.0%)	2.069	0.150
NYHA functional class				2.542	0.468
I	1 (1.4%)	0 (0%)	1 (4%)		
II	39 (56.5%)	24 (54.5%)	15 (60%)		
III	25 (36.2%)	17 (38.6%)	8 (32.0%)		
IV	4 (5.8%)	3 (6.8%)	1 (4.0%)		
History of LSVS	67 (97.1%)	43 (97.7%)	24 (96.0%)	0.169	0.681
History of TVP	16 (23.2%)	11 (25.0%)	4 (16.0%)	0.759	0.384
Atrial fibrillation	60 (87.0%)	39 (88.6%)	21 (84.0%)	0.302	0.583
Pacemaker implantation	2 (2.9%)	1 (2.3%)	1 (4.0%)	0.169	0.681
Laboratory findings					
Creatinine (µmol/L)	68.2 ± 23.1	68.3 ± 20.2	68.0 ± 28.0	0.050	0.961
BUN (mmol/L)	6.4 (4.9, 8.3)	5.9 (4.9, 8.0)	6.5 (4.9, 8.3)	0.406	0.685
Total bilirubin (µmol/L)	22.4 ± 9.9	23.5 ± 10.6	20.5 ± 8.3	1.228	0.224
ALT (U/L)	18.0 (13.0, 23.5)	17.0 (13.0, 21.0)	18.0 (15.0, 27.0)	0.563	0.574
AST (U/L)	31.5 (25.0, 39.0)	30.0 (25.0, 39.0)	33.0 (25.0, 41.0)	0.108	0.914
Transthoracic echocardiography					
LVEF (%)	62.8 ± 6.2	62.9 ± 7.1	62.8 ± 4.2	0.041	0.967
PAP (mmHg)	41.1 ± 10.6	41.9 ± 11.4	39.6 ± 9.1	0.847	0.400
ULDRV (mm)	61.0 ± 8.6	60.7 ± 8.4	61.3 ± 9.3	0.274	0.785
ULDRA (mm)	83.0 ± 16.6	87.7 ± 16.3	75.3 ± 14.4	3.166	0.002
Preoperative TR jet area (${\mathrm{cm}}^{2}$)	23.5 ± 9.1	25.3 ± 10.1	20.2 ± 5.9	2.279	0.026
Postoperative TR jet area (${\mathrm{cm}}^{2}$)	4.2 ± 3.4	4.5 ± 3.6	3.5 ± 2.9	1.202	0.234

BMI, body mass index; BUN, blood urea nitrogen; AST, aspartate aminotransferase; ALT, alanine aminotransferase; LVEF, left ventricular ejection fraction; ULDRV, upper and lower diameter of the right ventricle; ULDRA, upper and lower diameter of the right atrium; PAP, Pulmonary arterial pressure; LSVS, left-side valve surgery; TVP, tricuspid valvuloplasty; TR, tricuspid valve regurgitation; ePTFE, expanded Polytetrafluoroethylene; NYHA, New York Heart Association.

### 2.2 Procedure

Traditional TVP was usually performed by median sternotomy with the heart in a state of arrest. However, patients with severe TR following cardiac surgery often had organ dysfunction due to long-term systemic circulation disorders, and the operative mortality rate is as high as 8.8% [16]. The new surgical method we had reported before could effectively avoid atrioventricular block and achieve good surgical results [11].

All patients were placed with a double lumen tracheal intubation, and only the left lung was ventilated to increase the exposure of the surgical field of vision. In the early stage, extracorporeal circulation is established through superior and inferior vena cava catheterization. As the drainage tube of the superior vena cava, the anaesthetist inserted the 16F artery catheter through the internal jugular vein to reduce the amount of blood returning. Then, the right femoral vein cannula (24F to 28F) was placed as the inferior vena cava drainage tube, subsequently, the appropriate femoral artery cannula (17F to 19F) was selected according to the patient’s body surface area and implanted through the right femoral artery. In the later stage, all patients were used a single tube drainage method, with no catheterization of the superior vena cava, only femoral vein catheterization (usually 26F or 28F), and negative pressure suction. A 3- to 4- centimetre skin incision was made below the right nipple for males and inferior sulcus for females. Two separate working ports were performed in the fourth and the fifth intercostals. After opening the chest cavity, both the pericardium and right atrium were incised simultaneously. When the leaflet area decreased due to leaflet retraction and heart enlargement led to relatively small valve leaflets, leaflet patch augmentation technology was needed. The extent of the incision of the tricuspid valve leaflets depended on the condition of the tricuspid valve lesion. If the anterior and posterior leaflets of the tricuspid valve were thickened and curled significantly, the incision area should be larger. A wide curved incision should be made along the root of the leaflet from the anterior septal junction to the posterior septal junction. The width and length of the deficit created was measured with the edge of the anterior leaflet, under enough traction to meet the septal annulus. A patch was cut to measure the width + 5 mm and length + 5 mm. The patch was then sutured to the leaflet resection margin and annulus and would serve as the main part of the valve, and the natural valve leaflet would turn to the right ventricle to play the role of commissure area and partially extended the chord. Other repair techniques have also been applied as needed, such as commissurotomy, artificial chordae, and prosthetic ring implantation. Throughout the procedure, all operations were performed on beating heart with normothemic cardiopulmonary bypass.

### 2.3 Statistical Analysis

The data were statistically analysed using SPSS 27.0 (version 27.0; SPSS Inc., Chicago, IL, USA), and the data were tested for normal distribution, with *t*-test for measures that conformed to normal distribution, expressed as mean ± standard deviation, and non-parametric test (Mann-Whitney U) for measures that did not conform to normal distribution, expressed as quartiles [Q50 (Q25, Q75)]; Count data were expressed using chi-square test and frequency (%). A difference of *p* < 0.05 was used to indicate statistical significance. Kaplan-Meier analysis was used to evaluate freedom from cardiac death and reoperation.

## 3. Results

### 3.1 Perioperative

Except for three deaths (4.3%), all others were discharged smoothly. Three deceased patients all experienced multiple organ failure during the perioperative period, one of which in the BP group was caused by low cardiac output and two in the ePTFE group by low cardiac output and acute respiratory dysfunction syndrome respectively. The remaining underwent echocardiography before discharge, and the TR jet area decreased from 25.3 ± 10.1 to 4.5 ± 3.6 ${\mathrm{cm}}^{2}$ in the BP group and 20.2 ± 5.9 to 3.5 ± 2.9 ${\mathrm{cm}}^{2}$ in the ePTFE group after surgery. Although the degree of TR was significantly relieved compared with that before the operation, three patients (6.8%) in the BP group and one patient (4.0%) in the ePTFE group were discharged with severe TR, which may be associated with preoperative TR jet area and significantly enlarged hearts. Echocardiography of two patients (2.9%) showed that the blood flow velocity at the tricuspid valve was accelerated, and the flow velocity reached 1.9 m/s in the BP group and 1.8 m/s in the ePTFE group. The average transvalvular pressure difference of these two patients was estimated to be 9 mmHg and 6 mmHg, respectively, and the anterior lobe was slightly lengthy. Perioperative data and complications are shown in Table 2.

**Table 2.** **Postoperative complications**.

Variable	Entire study Population	Bovine pericardial group	ePTFE group	t/χ^2^	*p*
	N = 69	N = 44	N = 25		
CPB (min)	137.5 ± 31.3	141.5 ± 34.0	130.5 ± 25.0	1.407	0.164
Ventilator-assisted breathing (h)	13.5 (8.0, 25.0)	13.0 (8.0, 26.5)	14.0 (8.5, 19.5)	0.219	0.827
ICU (h)	53.5 (42.0, 98.0)	57.0 (44.5, 113.0)	50.0 (33.5, 92.5)	1.343	0.179
Postoperative 24 h chest drainage fluid (mL)	170.0 (100.0, 280.0)	180.0 (95.0, 325.0)	150.0 (110.0, 232.5)	0.929	0.353
Low cardiac output (n)	8 (11.6%)	7 (15.9%)	1 (4.0%)	2.206	0.137
Exploration of thoracotomy (n)	3 (4.3%)	1 (2.3%)	2 (8.0%)	1.257	0.262
Respiratory insufficiency (n)	6 (8.7%)	4 (9.1%)	2 (8.0%)	0.024	0.877
CRRT (n)	4 (5.8%)	3 (6.8%)	1 (4.0%)	0.232	0.630
Perioperative mortality (n)	3 (4.3%)	1 (2.3%)	2 (8.0%)	1.257	0.262

CPB, cardiopulmonary bypass; ICU, intensive care unit; CRRT, Continuous Renal Replacement Therapy; ePTFE, expanded Polytetrafluoroethylene.

### 3.2 Follow-up

**Fig. 1.**
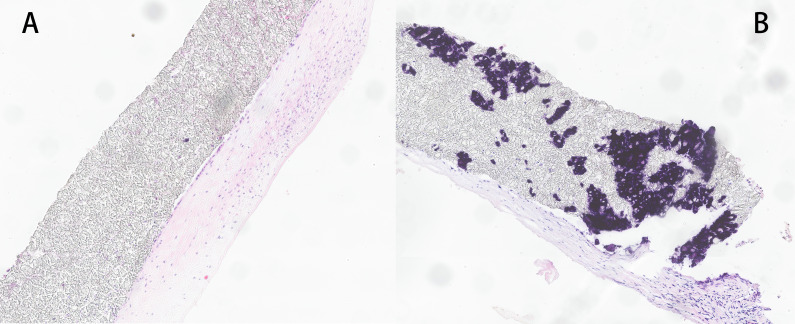
**Pathological examination of ePTFE**. (A) Collagen coverage on the surface of the patch. The magnification: 7.2X. (B) Scattered calcifications exist on the patch. The magnification: 7.2X. ePTFE, expanded Polytetrafluoroethylene.

**Fig. 2.**
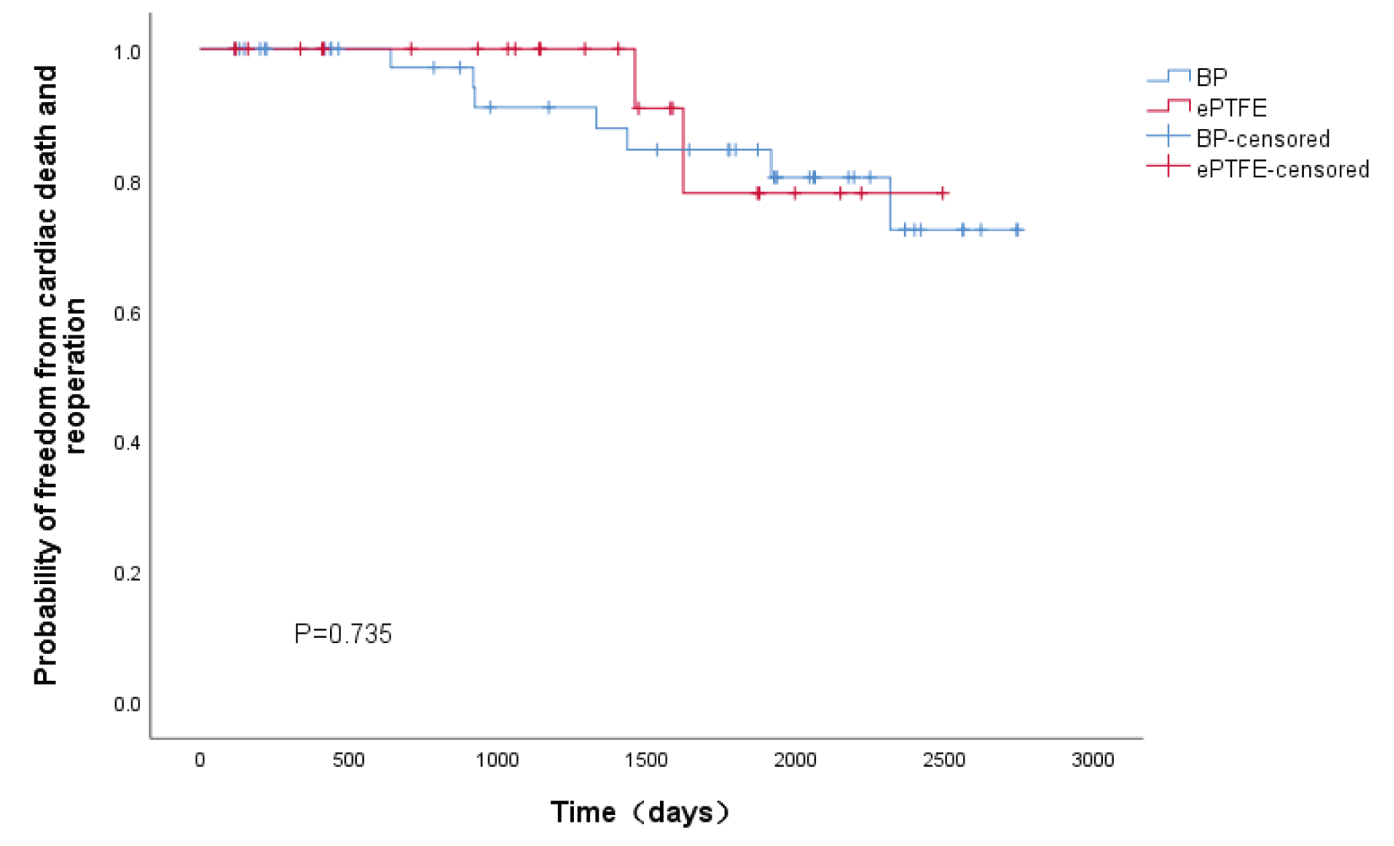
**Kaplan-Meier survival curve and comparison between bovine pericardium and ePTFE group**. BP, bovine pericardium; ePTFE, expanded Polytetrafluoroethylene.

All patients received complete outpatient or telephone follow-up, with an average follow-up time of 50.1 ± 24.3 months (from 2 months to 7 years and 10 months). In the BP group and the ePTFE group, there was one case each of reoperation for severe TR or stenosis. In the BP group, significant shortening of the chordae below the tricuspid septum and adhesion of the leaflet was seen; in the ePTFE group, fibrous hyperplasia was seen on the surface of the patch, and the leaflet was stiff and poorly mobile, and the pathology showed multiple scattered calcifications of the patch, with collagenous coverage seen on the surface (Fig. 1). Among the four patients who were discharged with severe TR (5.8%), one of whom was reoperated in BP group, and the degree of TR in the remaining three cases was lower than that at discharge, but one patient in the BP group still needed diuretics to control ascites. During the follow-up period, there were 7 cases of severe TR (10.1%), including 5 in the BP group (11.4%) and 2 in the ePTFE group (8.0%); there were 5 cases of tricuspid stenosis (7.2%), including 4 in the BP group (9.1%) and 1 in the ePTFE group (4.0%), with ultrasonographic indications of rigidity of anterior leaflet activity and poor motility in the BP group, and the ePTFE group was the reoperation patient mentioned previously; and a total of 13 patients (18.8%) needed diuretics due to lower limb oedema, including 9 (20.5%) in the BP and 4 (16.0%) in the ePTFE group. As of this follow-up, there were 6 deaths (8.7%), including 5 in the BP group (11.4%) and 1 in the ePTFE group (4.0%). The cause of death was heart failure in 5 cases and acute myocardial infarction in 1 case. The performance of the different materials used for augmentation of the anterior tricuspid valve leaflet was evaluated. The Kaplan–Meier curves in Fig. 2 show no significance of the two groups in terms of freedom from cardiac death and reoperation.

## 4. Discussion

Totally endoscopic TVP on beating heart with normothemic cardiopulmonary bypass was a safe and repeatable technology, which can effectively reduce the degree of TR and restore normal or near-normal function. However, it was often difficult to achieve ideal results by relying only on annuloplasty. Patches need to be added to reconstruct the valve. We used BP patch and ePTFE patch to augment the leaflets of tricuspid valve, which has achieved good early results [17]. However, in the long-term follow-up, four patients in the BP group were found to have stenosis of the tricuspid valve orifice, stiff leaflet movement, and poor mobility; and one patient in the ePTFE group had reoperation for calcification of the patch.

For pericardial patches, Quinn *et al*. [18] reported good repair results, following up 60 patients who underwent mitral valve reconstruction with fresh autologous pericardium. They found good hemodynamic results, no long-term patch stiffness or calcification, and no patch contracture. However, Myers *et al*. [19] reported the opposite result. The BP fixed with glutaraldehyde worsened due to calcification, and the fresh autologous pericardium showed inflammatory cell infiltration and different degrees of contraction. In other studies, it was also found that the autologous or heterogeneous pericardium treated with glutaraldehyde as a leaflet patch had patch degeneration or calcification [13, 20]. In contrast to the pericardial patch, the ePTFE patch performed well in reconstructing the right ventricular outflow tract, and there was no obvious stenosis, calcification, or pulmonary embolism in the long-term results [21, 22, 23, 24, 25]. However, in the repair of the aortic valve, it was found that it was easy to tear in the repair of the unicuspid aortic valve [15], while it performed well in the repair of the tricuspid aortic valve [26], which may be related to the high stress on the unicuspid aortic valve structure [27].

Many studies on valve patches focus on the left-sided valve and right ventricular outflow tract, fewer studies focus on the tricuspid valve. We used BP and ePTFE patches to augment the tricuspid valve. Only two patients showed mild stenosis in the early postoperative period, which may be related to the inappropriate patch size. TR was still severe in 4 cases, which may be related to the higher pulmonary artery pressure and significantly enlarged right heart. This also suggested that surgery should be performed before significant cardiac remodelling occurred to achieve satisfactory results. During a long follow-up, 4 cases of tricuspid stenosis with stiff leaflet movement, poor motion, and leaflet contracture were found in the bovine pericardial patch group. In the polytetrafluoroethylene group, no avulsion was seen, which may be related to the tricuspid valve being on the low-pressure side of the circulation, but there was one case of reoperation due to calcification of the leaflets and stiffness of movement leading to stenosis and regurgitation.

As reported by others, pericardial patches have a high tendency of calcification during the first 5–8 years or other pathological changes, such as thickening and curling [12, 13, 28, 29], but in our study, some patients used it for less than this time. Therefore, we look forward to longer follow-up results. As a synthetic material, ePTFE is at risk of tearing in aortic valve leaflet enlargement [15], and its surface micropore structure provides lesions for dystrophic calcification [30], as well as a tendency to stiffen leaflet movement when covered with collagenous tissue. The application results of the two types of patches were dissatisfactory, so more new material patches were needed to be studied. For example, the extracellular matrix from the small intestinal submucosa has been used as a patch for cardiovascular reconstruction in children and has shown advantages [31, 32].

The ideal implant material should have lasting flexibility, withstand the ventricular wall stress of multiple cardiac cycles, and should not cause any adverse immune reaction, fibrosis or calcification, any of which will inhibit the recovery of surrounding tissues and therefore avert the goal of natural phenotype and normal function.

## 5. Conclusions

In patients with TR after LSVS, augmentation of the anterior tricuspid leaflet achieved satisfactory results. However, the durability of the patch was unsatisfactory, with a risk of reduced flexibility and stiffness of movement for the BP patch and a risk of calcification for the ePTFE patch.

## Data Availability

The raw data supporting the conclusions of this article will be made available by the authors, without undue reservation.
